# Perceptions of General Dental Practitioners Regarding Do-It-Yourself Aligners: A Cross-Sectional Survey in East Bangalore

**DOI:** 10.7759/cureus.111775

**Published:** 2026-06-29

**Authors:** Aishwarya Dixith S, Chaitra K R, Veekshith M, Tarulatha R Shyagali

**Affiliations:** 1 Department of Orthodontics, MR Ambedkar Dental College and Hospital, Bangalore, IND

**Keywords:** bangalore, dentists, diy aligners, orthodontics, perception, questionnaire study

## Abstract

Objectives

To assess the perception of general dental practitioners in East Bangalore regarding the concept of do-it-yourself (DIY) aligners.

Materials and methods

A cross-sectional questionnaire-based survey was conducted among 114 general dental practitioners practicing in East Bangalore. A validated electronic questionnaire comprising 19 questions was distributed online. Descriptive statistics were used to summarize responses, and comparisons between practitioners familiar and unfamiliar with DIY aligners were performed using the Chi-square test. Statistical significance was set at p<0.05.

Results

Among the 114 participants, 59 (51.8%) were familiar with the concept of DIY aligners, while 55 (48.2%) were unfamiliar. Regarding the primary purpose of DIY aligners, 66.1% of familiar practitioners and 41.8% of unfamiliar practitioners identified correction of minor tooth movements such as spacing and mild crowding as the main indication. Significant differences were observed between the two groups in their perception of the primary purpose of DIY aligners (p=0.02). Practitioners familiar with DIY aligners were more likely to report having encountered patients who used DIY aligners without professional guidance (42.4% vs. 14.5%; p=0.001) and to have observed complications arising from unsupervised use (37.3% vs. 16.4%; p=0.02). Significant differences were also found regarding the frequency of patient inquiries about DIY aligners (p<0.001) and perceived cost-effectiveness (p=0.04).

Conclusion

General dental practitioners demonstrated varying levels of familiarity with DIY aligners. Practitioners familiar with the concept were more likely to encounter patients using DIY aligners and to report complications associated with their unsupervised use. Overall, concerns regarding potential risks highlight the importance of professional supervision in orthodontic treatment.

## Introduction

Orthodontics has evolved from a specialist-driven field to include general dental practitioners and, more recently, direct-to-consumer (DTC) clear aligner systems. Marketed as affordable and convenient, DTC aligners allow patients to undergo treatment without direct clinical supervision, often appealing through online promotions emphasizing aesthetics and convenience. However, the absence of comprehensive clinical examinations, radiographs, and professional monitoring can lead to complications such as malocclusion, temporomandibular joint disorders, root resorption, and periodontal damage. Furthermore, risk disclosures on many DTC websites are often inadequate, limiting informed consent [1].

In India, the Indian Orthodontic Society (IOS) has opposed unsupervised aligner therapy through initiatives such as the Smile Rally and has urged the Dental Council of India (DCI) to regulate direct aligner sales and at-home dental scans. Similar concerns have been raised globally by organizations including the American Association of Orthodontists, the American Dental Association, and the British Orthodontic Society [2].

Studies and patient reports have highlighted adverse outcomes associated with DTC aligners, including gum inflammation, bite problems, poor aftercare, and irreversible oral damage [3]. These concerns underscore issues related to patient safety, ethics, and regulation. Despite dentists playing a crucial role in safeguarding oral health through evidence-based care and patient education, limited research has explored Indian dentists' perceptions of DIY aligners, particularly regarding their safety, effectiveness, and ethical implications in urban settings such as East Bangalore [4].

The aim is to evaluate the perceptions of general dental practitioners regarding the efficacy, potential adverse effects, and clinical recommendations associated with do-it-yourself (DIY) orthodontic aligners in the context of recent advancements and shifts in the orthodontic care landscape.

The objective of this study was to assess the awareness of general dental practitioners regarding the clinical efficacy and limitations of DIY aligners, evaluate their perceptions of the potential risks and adverse effects associated with unsupervised orthodontic treatment, explore their recommendations for the safe and ethical use of aligners, including the need for regulatory measures, and determine how these perceptions may help orthodontists develop strategies to maintain competitiveness while ensuring patient safety in the evolving orthodontic market.

## Materials and methods

A descriptive, cross-sectional, questionnaire-based survey was conducted among general dental practitioners practicing in East Bangalore to assess their perceptions regarding DIY orthodontic aligners.

Study population and sampling

The target population comprised general dental practitioners currently practicing in East Bangalore. A total of 114 practitioners participated in the survey during November 2023. Participation was voluntary, and only one response per participant was accepted to avoid duplication, with restrictions applied to allow a single submission per unique email address.

Questionnaire development and validation

A structured electronic questionnaire consisting of 19 closed-ended and multiple-choice questions was designed to evaluate participants’ awareness, perceived efficacy, potential side effects, and recommendations related to DIY aligners. The questionnaire underwent content validation by a panel of experienced orthodontists to ensure clarity, relevance, and comprehensiveness. Modifications were incorporated based on expert feedback prior to dissemination.

Data collection

The validated questionnaire was distributed electronically via email and online Google survey platforms. Respondents were required to provide a single response using their registered email ID. The survey link remained active for a fixed period to maximize response rates while maintaining data integrity.

Ethical considerations

The study adhered to ethical guidelines for survey research. Informed consent was obtained electronically prior to participation, and anonymity and confidentiality of responses were maintained.

Study design and location

A cross-sectional questionnaire-based survey was conducted among general dental practitioners practicing in East Bangalore, Karnataka, India, during November 2023.

Study duration

The study was conducted over a period of one month (November 2023).

Sampling technique

Participants were selected using a simple random sampling technique. The validated electronic questionnaire was distributed online, and only one response per email ID was permitted to avoid duplicate entries. 

Inclusion and exclusion criteria

The study included general dental practitioners currently practicing in East Bangalore who were willing to participate, provided informed consent, and had access to the online questionnaire. Orthodontists and other dental specialists were excluded from the study. In addition, incomplete questionnaire responses, duplicate submissions, and responses from practitioners not currently practicing in East Bangalore were excluded from the final analysis.

Ethical considerations

The study protocol was reviewed and approved by the Institutional Ethics Committee (IEC) of MR Ambedkar Dental College and Hospital, Bengaluru, before commencement of the study. Informed consent was obtained electronically from all participants. The confidentiality and anonymity of the respondents were maintained throughout the study.

Statistical analysis

Statistical Package for the Social Sciences (SPSS) for Windows, Version 22.0 (released 2013; IBM Corp., Armonk, NY) was used to perform the statistical analyses. Descriptive analysis of all the explanatory variables, i.e., responses to the study questionnaire, was performed using frequencies and proportions. The Chi-square test was used to compare the distribution of participants' responses to the study questionnaire based on their familiarity with the concept of DIY aligners. The level of significance was set at p<0.05.

## Results

The sociodemographic characteristics of the study participants are summarized in Table 1. The study population (n=114) was predominantly composed of young adults, with 87.7% between 20 and 30 years of age. This indicates that the study sample largely comprised individuals in their early professional or adult life stage, while older age groups were underrepresented, with only 1.8% aged 50-60 years. With respect to gender distribution, the sample showed a marked predominance of females (77.2%) compared to males (22.8%). This skewed representation suggests either higher participation rates among women or a study setting where females constitute the majority (e.g., healthcare, teaching, or academic environments). In terms of nationality, the participants were overwhelmingly Indian (97.4%), with only a small fraction (2.6%) being non-Indian. This reflects the localized nature of the study setting and indicates that the findings may be most generalizable to an Indian population (Table 1).

**Table 1:** Distribution of sociodemographic characteristics among study participants

Variable	Category	n	%
Age	20-30 yrs	100	87.70%
	30-40 yrs	9	7.90%
	40-50 yrs	3	2.60%
	50-60 yrs	2	1.80%
Gender	Males	26	22.80%
	Females	88	77.20%
Nationality-Indian	Yes	111	97.40%
	No	3	2.60%

The majority of participants were relatively early in their professional careers, with 85.1% having less than five years of practice experience. Only a small proportion reported longer durations, with 5.3% each practicing for 5-10 years or more than 15 years, and 4.4% practicing for 10-15 years. This indicates that the study sample was predominantly composed of younger professionals at an early stage of their clinical careers.

In terms of the patient population managed over the past five years, a significant proportion (67.5%) had attended to fewer than 1000 patients, which aligns with their shorter duration of professional practice. A smaller fraction reported higher patient loads, with 15.8% treating 1000-2000 patients, 6.1% treating 2000-3000 patients, and 10.5% treating more than 3000 patients during the same timeframe (Table 2).

**Table 2:** Distribution of professional practice characteristics among study participants

Variable	Category	n	%
Years in practice	<5 yrs	97	85.1%
	5-10 yrs	6	5.3%
	10-15 yrs	5	4.4%
	>15 yrs	6	5.3%
Patient population from past 5 years	<1000	77	67.5%
	1000-2000	18	15.8%
	2000-3000	7	6.1%
	>3000	12	10.5%

Table 3 summarizes the distribution of participants' responses to the study questionnaire. Among the participants, 51.8% were familiar with DIY aligners, while 48.2% were not. Over half (54.4%) perceived aligners as useful for correcting minor tooth movements, with fewer identifying general alignment (21.9%) or relapse prevention (20.2%) as their primary purpose. Patient inquiries about DIY aligners were reported at least occasionally by 63.2% of participants, though only 27.2% reported frequent inquiries. Perceptions of effectiveness were cautious, with 50.0% rating them as somewhat effective, 15.8% as highly effective, and 23.7% being unsure. Clinical encounters with DIY aligner users were reported by 28.9% of practitioners. The main concerns expressed were potential harm to oral health (44.7%), misalignment (27.2%), and poor fit (15.8%).

**Table 3:** Distribution of participants' responses to the study questionnaire DIY, do-it-yourself

Variable	Category	n	%
Are you familiar with the concept of DIY aligners?	Yes	59	51.80%
	No	55	48.20%
In your opinion, what is the primary purpose of dental aligners?	Correcting minor movements like spacing, mild crowding	62	54.40%
	Alignment of teeth	25	21.90%
	Preventing relapse after orthodontic treatment	23	20.20%
	Others	4	3.50%
How often do patients inquire about or mention DIY aligners during consultations?	Frequently	31	27.20%
	Occasionally	41	36.00%
	Rarely	25	21.90%
	Never	17	14.90%
What is your general perception of DIY aligners in terms of their effectiveness?	Highly effective	18	15.80%
	Somewhat effective	57	50.00%
	Ineffective	12	10.50%
	Not sure	27	23.70%
Have you encountered patients who have used or attempted to use DIY aligners without professional guidance?	Yes	33	28.90%
	No	81	71.10%
What concerns, if any, do you have regarding the use of DIY aligners without professional supervision?	Poor fit	18	15.80%
	Misalignment	31	27.20%
	Potential harm to oral health	51	44.70%
	Others	14	12.30%
Do you believe that DIY aligners can serve as a cost-effective alternative for some patients?	Yes	37	32.50%
	No	36	31.60%
	Not sure	41	36.00%
In your practice, do you provide patients with information or recommendations regarding DIY aligners?	Yes, regularly	22	19.30%
	Occasionally	39	34.20%
	No	53	46.50%
If a patient has already used a DIY retainer, how do you approach the situation during a consultation?	Discuss potential issues and recommend professional evaluation	71	62.30%
	Provide guidance on proper usage	27	23.70%
	Others	16	14.00%
Are there specific resources or educational materials you recommend to patients interested in DIY aligners?	Yes	39	34.20%
	No	44	38.60%
	Not applicable	31	27.20%
In your view, what role should general practitioners (Dental) play in guiding patients on the use of DIY aligners and Orthodontist-supervised aligners?	Essential guidance and supervision	53	46.50%
	Provide information but allow patient choice	41	36.00%
	Limited involvement	10	8.80%
	Others	10	8.80%
In your experience, have you encountered any complications or issues arising from patients using DIY aligners without supervision?	Yes	31	27.20%
	No	46	40.40%
	Not sure	37	32.50%

Opinions on cost-effectiveness were divided: 32.5% agreed, 31.6% disagreed, and 36.0% were unsure. Most practitioners reported not providing information on DIY aligners (46.5%), while 34.2% did so occasionally. When patients had already used DIY retainers, the majority (62.3%) discussed potential risks and recommended professional evaluation. Regarding professional roles, 46.5% supported essential guidance and supervision, while 36.0% preferred providing information while allowing patient choice. Complications related to DIY aligners were reported by 27.2%, with 40.4% denying such experiences and 32.5% being uncertain about them (Table 3).

As shown in Table 4, participants’ familiarity with DIY aligners significantly influenced several responses. Those familiar with the concept were more likely to recognize their role in correcting minor tooth movements (66.1% vs. 41.8%, p=0.02) compared to unfamiliar participants, who more often associated aligners with relapse prevention (29.1% vs. 11.9%). Familiar participants also reported a greater frequency of patient inquiries, with 40.7% noting frequent inquiries compared to only 12.7% among the unfamiliar (p<0.001). Experience with patients who had attempted DIY aligners without supervision was also more common in the familiar group (42.4% vs. 14.5%, p=0.001). Similarly, complications associated with unsupervised DIY aligner use were reported significantly more often by familiar respondents (37.3% vs. 16.4%, p=0.02). Cost-effectiveness perceptions differed as well, with familiar participants more often disagreeing that DIY aligners were cost-effective (40.7% vs. 21.8%, p=0.04). In contrast, no significant differences were observed between groups regarding perceived effectiveness of aligners (p=0.43), concerns about potential harm or misalignment (p=0.49), provision of patient information (p=0.43), approach to patients already using DIY retainers (p=0.38), or recommendations of educational resources (p=0.53). Similarly, views on the role of general practitioners in guiding patients did not differ significantly between groups (p=0.80) (Table 4).

**Table 4:** Comparison of participants' responses based on their familiarity with concepts of DIY aligners using the Chi-square test ^*^Chi-square test value DIY, do-it-yourself

Questions	Responses	Yes		No		ꭓ^2 ^Value	P-value
		n	%	n	%		
In your opinion, what is the primary purpose of dental aligners?	Correcting minor movements like spacing, mild crowding	39	66.10%	23	41.80%	9.522	0.02^*^
	Alignment of teeth	10	16.90%	15	27.30%		
	Preventing relapse after orthodontic treatment	7	11.90%	16	29.10%		
	Others	3	5.10%	1	1.80%		
How often do patients inquire about or mention DIY aligners during consultations?	Frequently	24	40.70%	7	12.70%	17.819	<0.001^*^
	Occasionally	23	39.00%	18	32.70%		
	Rarely	8	13.60%	17	30.90%		
	Never	4	6.80%	13	23.60%		
What is your general perception of DIY aligners in terms of their effectiveness?	Highly effective	8	13.60%	10	18.20%	2.766	0.43
	Somewhat effective	33	55.90%	24	43.60%		
	Ineffective	7	11.90%	5	9.10%		
	Not sure	11	18.60%	16	29.10%		
Have you encountered patients who have used or attempted to use DIY aligners without professional guidance?	Yes	25	42.40%	8	14.50%	10.717	0.001^*^
	No	34	57.60%	47	85.50%		
What concerns, if any, do you have regarding the use of DIY aligners without professional supervision?	Poor fit	11	18.60%	7	12.70%	2.417	0.49
	Misalignment	15	25.40%	16	29.10%		
	Potential harm to oral health	28	47.50%	23	41.80%		
	Others	5	8.50%	9	16.40%		
Do you believe that DIY aligners can serve as a cost-effective alternative for some patients?	Yes	18	30.50%	19	34.50%	5.088	0.04^*^
	No	24	40.70%	12	21.80%		
	Not sure	17	28.80%	24	43.60%		
In your practice, do you provide patients with information or recommendations regarding DIY aligners?	Yes, regularly	14	23.70%	8	14.50%	1.694	0.43
	Occasionally	20	33.90%	19	34.50%		
	No	25	42.40%	28	50.90%		
If a patient has already used a DIY retainer, how do you approach the situation during a consultation?	Discuss potential issues and recommend professional evaluation	37	62.70%	34	61.80%	1.915	0.38
	Provide guidance on proper usage	16	27.10%	11	20.00%		
	Others	6	10.20%	10	18.20%		
Are there specific resources or educational materials you recommend to patients interested in DIY aligners?	Yes	21	35.60%	18	32.70%	1.262	0.53
	No	20	33.90%	24	43.60%		
	Not applicable	18	30.50%	13	23.60%		
In your view, what role should general practitioners (Dental) play in guiding patients on the use of DIY aligners and Orthodontist-supervised aligners?	Essential guidance and supervision	30	50.80%	23	41.80%	1.01	0.8
	Provide information but allow patient choice	19	32.20%	22	40.00%		
	Limited involvement	5	8.50%	5	9.10%		
	Others	5	8.50%	5	9.10%		
In your experience, have you encountered any complications or issues arising from patients using DIY aligners without supervision?	Yes	22	37.30%	9	16.40%	7.521	0.02^*^
	No	18	30.50%	28	50.90%		
	Not sure	19	32.20%	18	32.70%		

## Discussion

Orthodontic practice has experienced a substantial transformation over the last decade with the rapid expansion of clear aligner therapy, DTC orthodontics, and DIY aligner systems. Traditionally, orthodontic treatment was performed exclusively under the supervision of orthodontists following comprehensive clinical examination, radiographic assessment, diagnosis, and individualized treatment planning. However, advancements in digital dentistry, social media marketing, teledentistry, and commercial aligner companies have changed patient attitudes toward orthodontic care. DIY aligner companies promote treatment primarily on the basis of affordability, convenience, and reduced need for regular dental appointments, making them attractive, particularly to younger adults and esthetically conscious patients. Despite these advantages, concerns regarding patient safety, inadequate diagnosis, and absence of clinical supervision continue to be widely discussed within the dental profession.

The present study was conducted to assess the perceptions of general dental practitioners in East Bangalore regarding the effectiveness, safety, and clinical implications of DIY aligners. The findings demonstrated that awareness of DIY aligners among practitioners was almost equally distributed, with nearly half of the participants being familiar with the concept. This indicates that DIY orthodontic systems are increasingly becoming part of routine dental discussions and patient inquiries. Similar observations were reported by Tabbaa et al. (2023), who found that public interest in DTC orthodontics is steadily increasing because patients perceive such systems as simpler and more convenient alternatives to conventional orthodontic treatment [5].

In the present study, the majority of practitioners considered DIY aligners to be only “somewhat effective,” especially for minor tooth movements such as mild spacing and crowding corrections. This finding is clinically relevant because orthodontic tooth movement is a biologically complex process requiring continuous monitoring and modification during treatment. Castroflorio et al. (2023) reported that the predictability of aligner-based tooth movement varies considerably depending on treatment design and complexity, indicating that unsupervised orthodontic therapy may not achieve reliable outcomes in moderate or severe malocclusions. Similarly, Chawla et al. (2024) observed that orthodontists generally consider clear aligners effective for selected tooth movements but less reliable for complex orthodontic corrections compared with conventional treatment modalities [6].

An important finding of the present study was that a large proportion of practitioners expressed concern regarding the potential harm caused by unsupervised DIY aligner therapy. Nearly half of the respondents identified oral health damage as the major concern associated with DIY orthodontics. This observation aligns with recent literature emphasizing the risks associated with orthodontic treatment performed without appropriate professional supervision. Adverse outcomes such as root resorption, gingival recession, periodontal breakdown, occlusal discrepancies, temporomandibular dysfunction, and worsening malocclusion have been associated with improperly monitored aligner therapy. A recent analysis by Wexler et al. (2023), evaluating adverse events related to DTC aligners, highlighted multiple complications resulting from inadequate diagnosis and the absence of in-person clinical assessment [7].

The present study also demonstrated that practitioners who were familiar with DIY aligners were significantly more likely to encounter patients who had attempted unsupervised orthodontic treatment. This reflects the increasing penetration of DTC orthodontic companies into mainstream dental awareness. Barhate et al. (2023) analyzed YouTube content related to DIY aligners and reported that social media platforms significantly influence patient perception and acceptance of unsupervised orthodontic treatment. The authors further noted that many online educational videos lacked scientific reliability and frequently underestimated the risks associated with self-directed orthodontics. Such findings highlight the growing influence of digital marketing and online platforms in shaping patient expectations regarding orthodontic care [8].

Interestingly, although some participants acknowledged that DIY aligners may provide a cost-effective alternative for selected patients, a considerable proportion remained uncertain regarding their economic benefits. This uncertainty may arise because complications associated with poorly supervised treatment frequently require corrective orthodontic or restorative procedures, ultimately increasing overall treatment costs. Recent reports regarding the shutdown of major DTC companies such as SmileDirectClub further emphasize the uncertainty and limitations associated with unsupervised orthodontic models. Orthodontists and professional organizations have consistently highlighted the importance of direct clinical supervision and comprehensive diagnosis in ensuring safe orthodontic outcomes [9].

Another important observation from the present study was that most practitioners preferred recommending professional orthodontic evaluation rather than encouraging unsupervised DIY aligner use. The majority believed that general dental practitioners should play an essential role in guiding patients toward orthodontist-supervised care. This reflects the ethical responsibility of dental professionals to prioritize patient safety and evidence-based treatment. Easson (2022) emphasized that dental professionals involved in DTC orthodontics must adhere to professional standards and ensure that patients are informed regarding the limitations and risks of remote orthodontic treatment [10].

The statistically significant association observed between familiarity with DIY aligners and awareness of complications suggests that practitioners with greater exposure to modern orthodontic trends are more capable of identifying potential risks and counseling patients appropriately. This finding underlines the importance of continuing dental education programs focusing on digital orthodontics, aligner therapy, and emerging trends in DTC dentistry. Cherian and Renji (2023) also highlighted the rapidly expanding aligner market in India and expressed concern regarding aggressive marketing strategies that may bypass professional orthodontic evaluation [11].

The findings of the present study are consistent with growing international concerns regarding the ethical, clinical, and medico-legal implications of DIY orthodontics. Although these systems may improve accessibility and convenience for selected individuals, they cannot replace comprehensive orthodontic diagnosis, radiographic analysis, periodontal assessment, and continuous professional supervision. Orthodontic tooth movement involves complex biological and biomechanical principles that require individualized treatment planning and monitoring to prevent irreversible complications [12].

Certain limitations of the present study should be acknowledged. This study was limited to general dental practitioners from East Bangalore, which may restrict the generalizability of the findings. The relatively small sample size and predominance of early-career practitioners may not fully represent the views of the broader dental community. As participation was voluntary and based on self-reported responses, selection bias, non-response bias, and reporting bias cannot be excluded. Additionally, the cross-sectional design captures perceptions at a single point in time and does not permit causal inferences. Further multicenter studies with larger and more diverse samples are recommended.

Despite these limitations, the present study provides valuable insight into the evolving perception of general dental practitioners regarding DIY aligners and DTC orthodontics. The findings emphasize increasing awareness among practitioners while simultaneously highlighting concerns regarding inadequate supervision, patient safety, and oral health complications. Orthodontists, educators, and policymakers may utilize these findings to strengthen public awareness programs, improve professional education, and reinforce the importance of evidence-based, professionally supervised orthodontic treatment.

## Conclusions

The findings of this study indicate that while a considerable proportion of general dental practitioners are familiar with the concept of DIY aligners, concerns remain regarding their safety, effectiveness, and lack of professional supervision. Most participants recognized the potential risks associated with unsupervised aligner therapy, including adverse effects on oral health and treatment outcomes. General practitioners were more likely to recommend referral to an orthodontist rather than endorse DIY aligners, emphasizing the importance of specialist involvement in the diagnosis, treatment planning, and monitoring of orthodontic care. Overall, the study highlights the need for increased awareness among both dental professionals and patients regarding the limitations and potential complications of DIY aligners. Orthodontic treatment should be carried out under appropriate professional supervision to ensure safe, predictable, and evidence-based outcomes.

## Appendices

Questionnaire

1. Years in practice 

A. Less than 5

B. 5-10

C. 10-15

D. 15-20

2. Gender 

A. Male 

B. Female 

C. Prefer not to say

3. Community size 

A. Metropolitan area 

B. Suburban/outside principal city 

C. Small city/town

4. Nationality - INDIAN

A. Yes

B. No

5. Patient population from the past 5 years

A. Less than 100

B. 100-500

C. 500-1000 

D. More than 1000

6. Are you familiar with the concept of do-it-yourself (DIY) aligners?

A. Yes

B. No

7. In your opinion, what is the primary purpose of dental aligners?

A. Correcting minor tooth movements like spacing, mild crowding

B. Preventing relapse after orthodontic treatment

C. Alignment of teeth

D. Other (please specify): _______________

8. How often do patients inquire about or mention DIY aligners during consultations?

A. Frequently

B. Occasionally

C. Rarely

D. Never

9. What is your general perception of DIY aligners in terms of their effectiveness?

A. Highly effective

B. Somewhat effective

C. Ineffective

D. Not sure

10. Have you encountered patients who have used or attempted to use DIY aligners without professional guidance?

a. Yes

b. No

11. What concerns, if any, do you have regarding the use of DIY aligners without professional supervision?

A. Poor fit

B. Risk of misalignment

C. Potential harm to oral health

D. Other (please specify): _______________

12. Do you believe that DIY aligners can serve as a cost-effective alternative for some patients?

A. Yes

B. No

C. Not sure

13. In your practice, do you provide patients with information or recommendations regarding DIY aligners?

A. Yes, regularly

B. Occasionally

C. No

14. If a patient has already used a DIY retainer, how do you approach the situation during a consultation?

A. Discuss potential issues and recommend a professional evaluation

B. Provide guidance on proper usage

C. Other (please specify): _______________

15. Are there specific resources or educational materials you recommend to patients interested in DIY aligners?

A. Yes

B. No

C. Not applicable

16. In your view, what role should dental general practitioners play in guiding patients on the use of DIY aligners and an Orthodontist’s supervision?

A. Essential guidance and supervision

B. Provide information but allow patient choice

C. Limited involvement

D. Other (please specify): _______________

17. In your experience, have you encountered any complications or issues arising from patients using DIY aligners without supervision?

A. Yes

B. No

C. Not sure

18. Any additional comments or insights regarding DIY aligners and their impact on dental practice?

Thank you for participating in this questionnaire! Your insights are valuable in understanding the perspectives of general dentists on the topic of DIY aligners.
